# SLE: Novel Postulates for Therapeutic Options

**DOI:** 10.3389/fimmu.2020.583853

**Published:** 2020-10-07

**Authors:** Kinga K. Hosszu, Alisa Valentino, Ellinor I. Peerschke, Berhane Ghebrehiwet

**Affiliations:** ^1^Department of Pediatrics, Memorial Sloan Kettering Cancer Center, New York, NY, United States; ^2^Department of Lab Medicine, Memorial Sloan Kettering Cancer Center, New York, NY, United States; ^3^The Department of Medicine, Stony Brook University, Stony Brook, NY, United States

**Keywords:** c1q, gC1qR, cC1qR, complement, SLE, novel hypothesis

## Abstract

Genetic deficiency in C1q is a strong susceptibility factor for systemic lupus erythematosus (SLE). There are two major hypotheses that potentially explain the role of C1q in SLE. The first postulates that C1q deficiency abrogates apoptotic cell clearance, leading to persistently high loads of potentially immunogenic self-antigens that trigger autoimmune responses. While C1q undoubtedly plays an important role in apoptotic clearance, an essential biological process such as removal of self- waste is so critical for host survival that multiple ligand-receptor combinations do fortunately exist to ensure that proper disposal of apoptotic debris is accomplished even in the absence of C1q. The second hypothesis is based on the observation that locally synthesized C1q plays a critical role in regulating the earliest stages of monocyte to dendritic cell (DC) differentiation and function. Indeed, circulating C1q has been shown to keep monocytes in a pre-dendritic state by silencing key molecular players and ensuring that unwarranted DC-driven immune responses do not occur. Monocytes are also able to display macromolecular C1 on their surface, representing a novel mechanism for the recognition of circulating “danger.” Translation of this danger signal in turn, provides the requisite “license” to trigger a differentiation pathway that leads to adaptive immune response. Based on this evidence, the second hypothesis proposes that deficiency in C1q dysregulates monocyte-to-DC differentiation and causes inefficient or defective maintenance of self-tolerance. The fact that C1q receptors (cC1qR and gC1qR) are also expressed on the surface of both monocytes and DCs, suggests that C1q/C1qR may regulate DC differentiation and function through specific cell-signaling pathways. While their primary ligand is C1q, C1qRs can also independently recognize a vast array of plasma proteins as well as pathogen-associated molecular ligands, indicating that these molecules may collaborate in antigen recognition and processing, and thus regulate DC-differentiation. This review will therefore focus on the role of C1q and C1qRs in SLE and explore the gC1qR/C1q axis as a potential target for therapy.

## C1q: A Brief Overview

The first component of complement, C1, is a multimeric protein comprised of C1q and the Ca^2+^ – dependent tetramer C1r_2_–C1s_2_ (1–6). C1q itself is a 460 kDa collagen-like glycoprotein that is comprised of six globular “heads” (gC1q) linked to six collagen-like “stalks” (cC1q), and serves as the recognition signal triggering the classical pathway of complement (7–9). Each subunit of C1q is made up of three different, but highly conserved polypeptide chains – A, B, and C (10, 11). C1q belongs to the collectin (collagen containing lectin) family of molecules that contain collagen-like sequences contiguous with non-collagen-like stretches. Although it lacks a consensus carbohydrate recognition domain (which allows other collectins to recognize glycoconjugates containing mannose and fucose on microorganisms but not on self-proteins), C1q contains collagen sequences which allow it to bind to protein motifs in immunoglobulin (Ig)G or IgM. These motifs allow C1q to bind to immune complexes and engage in complement-mediated microbial killing and phagocytosis (12–14). While the majority of C1q circulates in plasma, it is also synthesized by many cell types including macrophages and dendritic cells (DCs), and secreted locally at sites of inflammation (15–24). Approximately 80% of circulating C1q is associated with the C1 complex, while the remaining portion is in its monomeric, “free” form (25).

In recent decades multiple groups have shown evidence that C1q plays a role in recognizing and clearing altered self and apoptotic cells by binding to the apoptotic cell surface and initiating phagocytic uptake by macrophages and DCs through interaction with C1q receptors expressed both on the phagocytic cell, (e.g., cC1qR/CD91) and the apoptotic cell (gC1qR and phosphatidylserine) (26–29). This clearance of immune complexes and apoptotic debris is crucial for maintaining homeostasis to avoid immune recognition of hidden epitopes – a critical immunopathogenic event leading to autoimmune disease.

## C1q Receptors

C1q receptors mediate many immunologic functions involved in innate and adaptive immunity. There are at least two types of distinct, ubiquitously expressed cell surface molecules which bind human C1q: gC1qR, the receptor for the globular heads, and cC1qR, the receptor for the collagen tail (28, 30–35).

Predominantly found in the storage compartments of the endoplasmic reticulum, cC1qR (60 kDa), a homolog of calreticulin (CR) (sometimes also referred to as cC1qR/CR or the “collagen receptor”) fulfills a multiplicity of functions. It is a molecular chaperone, an extracellular compartment protein, an intracellular mediator of integrin function, an inhibitor of steroid hormone-regulated gene expression, and a receptor for C1q (36–43). However, studies have shown that C1q can only bind stably to cC1qR after it has been immobilized, heat-treated, or bound to IgG, suggesting that cC1qR is a receptor for an altered conformation of C1q (44, 45).

cC1qR does not contain a transmembrane domain or a GPI-anchor attachment site, and instead needs other adaptor molecules for signal transduction. One such molecule is CD91 (46), which binds to cC1qR and C1q on the surface of monocytes to initiate uptake of apoptotic cells (26). However, the uptake process cannot be completely inhibited by antibody blockade or genetic deficiency of CD91, indicating that it is not actually required for the C1q-mediated enhancement of phagocytosis (26, 47). Additional co-receptors of cC1qR are scavenger receptor A on antigen presenting cells (48), CD59 on neutrophils (49), α2β1 integrin and glycoprotein VI on resting platelets (50), MHC class I on T cells (51), and CD69 on human peripheral blood mononuclear cells (PBMCs) (52).

GC1qR (p32/p33/HABP1) is another well-described C1q receptor. It is a highly acidic homotrimer, comprised of three 33-kDa chains with a ubiquitous and multi-compartmental distribution including on the cell surface. As a result, gC1qR has a highly asymmetric surface charge with a negatively charged “solution face” exposed to plasma and a neutral or basic “membrane face” on the reverse side, suggesting that the two sides have different functions (53–56). It is present on the surface of human monocytes, DCs, macrophages, and many other cells (19, 33, 34, 57, 58). Additionally, gC1qR’s capacity to elicit biological responses and transduce intracellular signals affects a variety of cell types (32, 57, 59–64). Similar to cC1qR, it lacks a transmembrane segment, and requires a docking/signaling partner, some of which are β1-integrins on endothelial cells (32), vasopressin V2 receptor on the HEK 293 cell line, alpha(1B)-adrenergic receptor on the COS 7 cell line (65), DC-SIGN on DCs (66, 67) and LAIR-1 on DCs and T cells (68–71).

Due to gC1qR’s ability to recognize and bind to a plethora of ligands, many pathogens employ immune escape mechanisms to exploit the normal regulatory functions of C1q/gC1qR. Among the growing list of pathogenic microorganisms are HIV (67, 72–74), adenovirus (75, 76), Epstein-Barr virus (77), Herpesvirus Saimiri (78), rubella virus (79–81), hepatitis B virus (82), hepatitis C virus (HCV) (59, 63, 74), *L. monocytogenes* (83), *S. aureus* (84), and *B. cereus* (85). These microorganisms have a strong affinity for gC1qR, which further indicates that gC1qR plays an important role in immune regulation. For example, *in vitro* studies have shown that HCV, which binds gC1qR at the C1q binding site, employs gC1qR on monocyte-DC precursors to prevent DC immunogenic activity (57, 58).

## C1q and SLE

The connection between C1q and autoimmune diseases such as rheumatoid arthritis (RA) and systemic lupus erythematosus (SLE) is well established. In RA, antibodies to C1q may cross-react with collagen type II and contribute to the disease process that leads to tissue destruction and inflammation (86, 87). In animal models of RA, C1q function is impaired by autoantibodies, indicating a regulatory role for C1q in suppressing immune activity (87, 88). Moreover, a synthetic decapeptide corresponding to the A-chain of C1q injected into DBA/1 mice delays disease onset and reduces the severity of collagen-induced arthritis (86, 89).

Hereditary homozygous C1q deficiency, while rare, is the strongest known susceptibility factor for SLE (90–93). The vast majority of patients (≥95%) develop clinical symptoms closely related to SLE, with rashes, glomerulonephritis, and central nervous system disease (91, 94). Additionally, about a third of SLE patients have high affinity autoantibodies to C1q directed to a neo-epitope in the A-chain (91, 94). In a subset of patients who are C1q sufficient, the SLE disease process itself causes consumption of C1q, therefore mimicking the genetic deficiency of C1q. This acquired partial deficiency of C1q, either due to complement activation or to the presence of anti-C1q autoantibodies, is even more commonly observed in lupus patients than genetic C1q deficiency (92, 95, 96). Multiple studies have shown associations between the presence of anti-C1q antibodies and active nephritis in SLE (97–100). There is, however, evidence that the presence of anti-C1q antibodies is not associated with active lupus nephritis, but rather with SLE global activity, indicating that although C1q’s main function is the clearance of immune complexes during apoptosis, it has other biologic functions with inhibitory/protective factors (30).

C1q plays a critical role in recognizing harmful molecules, ranging from pathogen-associated molecular ligands (non-self) to damage-associated molecular targets (altered self) (29). Therefore, in this manner, C1q acts as a molecular bridge between the phagocytic cell and the apoptotic debris to be cleared. While many studies suggest that failure to properly clear apoptotic cells in the absence of C1q could result in an immunogenic state (91, 94, 101), many observations have challenged this idea. Disruption of other apoptotic uptake processes, such as those mediated by CD14 (102), β3 or β5 integrin (103), mannose-binding lectin (104), all result in the accumulation of apoptotic bodies without triggering autoimmunity. In fact, apoptotic cells can actively inhibit the inflammatory program. For example, preincubating macrophages with apoptotic cells can significantly reduce the inflammatory response induced by lipopolysaccharide (LPS) (105–107). During this process, anti-inflammatory cytokines, such as transforming growth factor (TGF)-β and interleukin (IL)-10, are released and act via paracrine or autocrine mechanisms to sustain an anti-inflammatory state (107). Administration or accumulation of apoptotic cells have been shown to ameliorate multiple inflammatory disorders, such as diabetes (108, 109), Experimental Autoimmune Encephalomyelitis (110, 111), arthritis (112), colitis (113), pulmonary fibrosis (114–116), fulminant hepatitis (117), contact hypersensitivity (118, 119), acute and chronic graft rejection (120–123), and hematopoietic cell engraftment (124–127). Data from these studies indicate that apoptotic cells modulate immune responses and can prevent the onset and/or establishment of inflammatory disease. Based on these observations, it is likely that processes other than the accumulation of apoptotic debris play a decisive role in SLE development.

In recent years, increasing evidence has emerged that aside from the recognition and triggering of the classical complement pathway, C1q also modulates the acquired immune response. In this context, C1q provides active protection from autoimmunity by silencing key molecular markers or regulating autoreactive immune cells.

Multiple studies have shown that C1q regulates cytokine secretion and polarizes antigen presenting cells (APCs) toward a tolerogenic phenotype (17, 128–135). Specifically, macrophages and DCs that have been exposed to C1q exhibit enhanced production of anti−inflammatory and reduced pro-inflammatory cytokines (129, 134, 135). Immature DCs (iDC) in the presence of immobilized C1q have reduced capacity to induce allogeneic Th1 and Th17 cells, and demonstrate a trend toward increased Treg proliferation (130, 136). Furthermore, C1q-primed macrophages have elevated PD-L1 and PD-L2 and suppressed surface CD40, and C1q-polarized DCs have higher surface PD-L2 and reduced CD86 (130). Plasmacytoid DCs (pDCs), a major interferon-α (IFN-α)-producing cell type, also play a pivotal role in SLE pathogenesis (137–139). In the presence of immune complexes, C1q interacts with pDCs and strongly inhibits IFN-α production (140–142), while in the absence of C1q, immune complexes can preferentially engage pDCs and increase IFN-α production (143). These data suggest that C1q provides a protective, anti-inflammatory function by regulating IFN-α production in pDCs.

Our lab was the first to show that monocytes are able to display macromolecular C1 on their surface with the globular heads of C1q displayed outwardly, toward the extracellular milieu (144). Thus, membrane associated C1q can potentially recognize and capture circulating immune complexes or pathogen-associated molecular patterns and signal monocytes to migrate into tissues, differentiate into macrophages or DCs, and initiate the process of antigen elimination. Unoccupied C1q, on the other hand, may silence key molecular players, ensuring that unwarranted DC-driven immune responses do not occur.

Using a C1q-deficient mouse model of SLE, Ling et al. showed that C1q ameliorates the response to self-antigens by modulating the mitochondrial metabolism of CD8+ T cells (145). Conversely, C1q deficiency can trigger an effector CD8+ T cell response to chronic viral infection leading to lethal immunopathology.

Taken together, these data suggest that upon interacting with APCs, C1q regulates the subsequent activation of T effector functions to modulate the adaptive immune response and prevent the initiation/propagation of autoimmunity.

## C1q Receptors as an Immune Checkpoint

**FIGURE 1:**
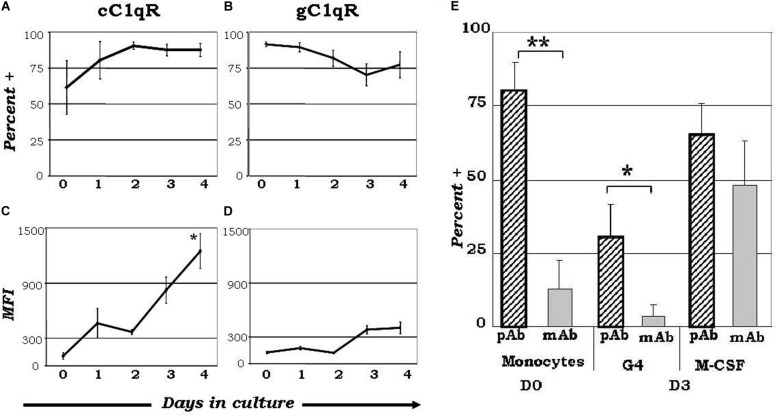
Varied expression of C1q receptors and specific binding orientation of surface bound C1q on monocyte-DC precursors may regulate DC differentiation events. Mononuclear cells cultured in the presence of GM-CSF+ IL-4 were analyzed for the expression of cC1qR **(A,C)** and gC1qR **(B,D)** expression, and C1q binding orientation **(E)**. **(A)** The percentage of cC1qR expression was variable on monocytes, but by day 2 nearly all monocyte-DCs had the receptor on their surface (*n* = 4). (B) On day 0, gC1qR was present on almost all the cells, and its expression was only slightly reduced by day 4 (*n* = 4). **(C)** Mean fluorescence analysis revealed that cC1qR expression was dramatically amplified by days 3 and 4 (*n* = 4). **(D)** Mean fluorescence intensity of gC1qR remained at relatively steady levels throughout the days (*n* = 4). **(E)** C1q is bound to the monocyte and DC surface via its globular head regions, while on M-CSF treated monocyte-macrophages its orientation is reversed. Binding orientation of C1q was determined using monoclonal antibodies specific to the globular head regions of C1q as well as polyclonal antibodies to the whole protein, and assessed by flow cytometry (*n* = 3). Experiments were gated on HLA-DR^+^ cells. *P < 0.05, **P < 0.01. [Adapted from ref (128)]

While the wide array of immunological processes exhibited by C1q appear to be the principal component of its immune-modulatory function, its underlying mechanisms remain poorly described. The unique structure of C1q, which allows it to interact with its primary receptors, gC1qR and cC1qR, via either its globular head or collagen tail domains, may shed light to this dilemma. The observation that C1q functions as a molecular switch during the narrow window of monocyte to DC transition (128, 133) is also reflected by the differential expression of gC1qR and cC1qR during this process (Figure 1) (128). While gC1qR is steadily expressed, the expression of cC1qR is low on monocytes and increases as the cells commit to the dendritic cell lineage. At the time corresponding to firm commitment to the DC lineage, there is an inverse correlation between gC1qR and cC1qR expression on the cell surface, which, in turn, may influence the nature and specificity of the cells’ response to C1q (128).

Upon binding to C1qR, specific pathways get activated to trigger downstream signaling. Incubating C1q or a monoclonal antibody which recognizes the C1q binding site on gC1qR, with T cells, inhibits T cell proliferation, possibly through the activation of PI3K, NADPH oxidase and p190 RhoGAP (53, 146). Additionally, it causes the inactivation of TC10, and the translocation of NKp44L from the cytoplasm to the plasma membrane (147). Ligand engagement of gC1qR at the C1q binding site (by HCV core protein and mAb) in LPS-stimulated monocytes increases PI3K activation and Akt phosphorylation, and in macrophages it induces A20 expression via P38, JNK and NF-κB signaling pathways, in an ERK independent manner (57, 58, 148). Similarly, engagement of gC1qR by C1q activates the MAPK and PI3K/AKT signaling pathways in macrophages (148). Furthermore, binding of HCV core protein to gC1qR down-regulates many inflammatory cytokines in macrophages, including IL-6 and IL-1β, indicating that gC1qR relays an anti-inflammatory signal (148). Conversely, ligation of cC1qR by a mAb increases TNFα and IL6 secretion, as well as the expression and phosphorylation of STAT6 in macrophages, indicating that cC1qR is a pro-inflammatory receptor (149).

C1q also engages in molecular complexing at the cell surface. In monocyte-derived iDCs, C1q, DC-SIGN and gC1qR form a trimolecular complex on the plasma membrane, which is presumed to modulate DC differentiation and function through DC-SIGN-mediated signaling pathways [26]. Signaling through DC-SIGN has been shown to increase phosphorylation of Raf-1 on Ser338 and Tyr340/341 (150). Furthermore, stimulation of DC-SIGN with a mannose receptor-1 Ab activates the MEK/ERK kinase cascade (151). However, whether direct stimulation of C1q participates in these signaling pathways still remains to be investigated.

The leukocyte-associated immunoglobulin-like receptor 1 (LAIR-1) is another C1q-binding transmembrane receptor that can serve as a potential co-receptor to gC1qR. On T cells, LAIR-1 engagement by C1q inhibits TCR signaling by decreasing the phosphorylation of LCK, LYN, ZAP-70, extracellular signal-regulated kinase, c-Jun N-terminal kinase 1/2, and p38, indicating that LAIR-1 activation may be a strategy for controlling inflammation (70). Studies by Son et al. showed that C1q and HMGB1 can cooperate to terminate inflammation, and induce the differentiation of monocytes to anti-inflammatory M2-like macrophages through a complex with RAGE and LAIR-1 (71). In myelomonocytes, the globular head of C1q binds to CD33 and LAIR-1 and activates CD33/LAIR-1 inhibitory motifs (68). Binding of C1q to LAIR-1 on monocytes significantly up-regulates the expression of IL-8, IL-10, LAIR-1, and the phosphorylation of JNK, p38-MAPK, AKT, and NF-κB (152).

Taken together, these data suggest that the regulatory effects of C1q may depend on specific C1q/C1qR interactions; and these interactions may in turn control the transition from the tolerogenic state toward a pro-inflammatory state. Fundamental to this mechanism is the differential expression of the C1q/C1qR system, which, through the engagement of distinct receptors (gC1qR versus cC1qR), and the resulting binding orientation of C1q – heads versus tails – actively avoids self-directed adaptive immune responses to modified-self as well as non-self antigens.

**FIGURE 2:**
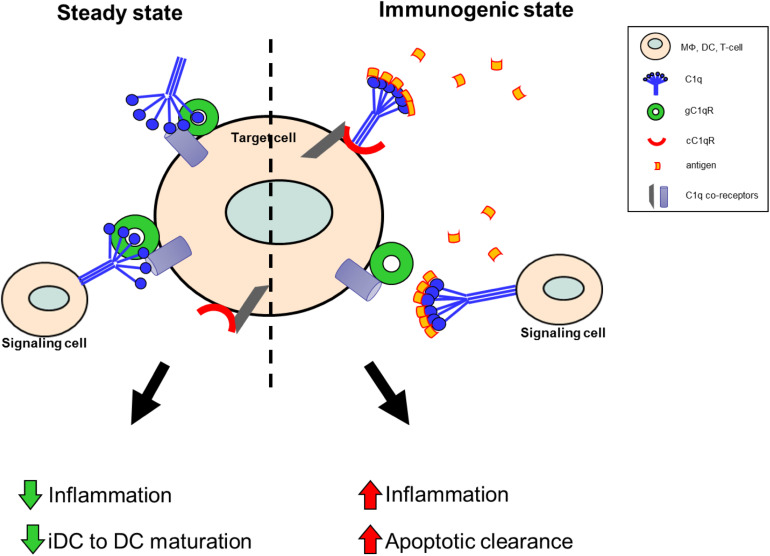
Theoretical model of the C1q/gC1qR immune checkpoint in inflammation and autoimmunity. Under steady state conditions, in the absence of danger signals (PAMPs, DAMPs, etc.), membrane-associated C1q on the signaling cell, or soluble C1q in the extracellular milieu, is available to bind to gC1qR on the target cell to support a tolerogenic state. During these conditions anti-inflammatory processes are dominant and DC maturation is decreased to keep cells in a tolerogenic/immature state. When C1q recognizes and captures circulating immune complexes or pathogen-associated molecular patterns, it undergoes a conformational change and only the collagen tail is available to bind. Thus, the resulting C1q/cC1qR interactions drive increased pro-inflammatory signals and signal monocytes to migrate into tissues, differentiate into macrophages or DCs, and initiate the process of antigen elimination.

As illustrated by Figure 2, this functional duality of the C1q/gC1qR axis is very similar to the role of the PD1/PDL1 checkpoint in cancer, which helps maintain the balance between immune surveillance and cancer cell proliferation (153). In this setting, the C1q/C1qR axis would serve as an immune checkpoint supporting a tolerogenic/anti-inflammatory signal by the interaction between membrane-associated C1q on the signaling cell or soluble C1q in the extracellular milieu, and the membrane associated C1q receptors on the target cell. Conversely, when this interaction is blocked by antigen binding to the soluble or membrane-associated C1q, a pro-inflammatory signal is relayed through cC1qR. These specific interactions ensure that the immune system is activated only at the appropriate time in order to minimize the possibility of chronic autoimmune inflammation.

## The C1q/C1qR Axis: A Functional Example

The role of C1q in the regulation of DC differentiation and function has been greatly studied in recent years. A significant portion of the work has centered, around the potential regulatory role of C1q during DC maturation, once the cells have fully committed to the DC lineage. These data show that C1q treatment of LPS-primed human iDCs decreases the cell surface expression of CD80, CD83 and CD86, the secretion of IL-6, TNF-α, and IL-10, as well as the ability of the cells to stimulate T helper (T_H_) 1 cell proliferation in a mixed leukocyte reaction (154). These results suggest that C1q treated iDCs may be resistant to LPS-induced maturation. Yamada and colleagues showed that C1q treatment after LPS-stimulation or CpG oligodeoxynucleotide induction suppresses IL-12p40 production in bone marrow-derived DCs, reduces NF-κB activity and delays the phosphorylation of p38, c-Jun N-terminal kinase, and extracellular signal-regulated kinase (155). These data further indicate that C1q may function by suppressing pro-inflammatory responses after DC activation. As ligation of gC1qR results in decreased secretion of pro-inflammatory cytokines like IL-6 and TNFα, soluble C1q in these experiments putatively acts through a gC1qR-mediated pathway.

However, in order to imitate the role of C1q as an opsonin *in vitro*, some studies employed immobilized C1q. Nauta and colleagues found that the uptake of C1q-opsonized apoptotic cells by iDCs stimulated the production of IL-6, IL-10, and TNF-α, without an effect on IL-12p70 (156). Additionally, iDCs placed on immobilized C1q, gC1q or cC1q, showed enhanced maturation, translocation of NF-κB to the nucleus and enhanced secretion of IL-12 and TNF-α, in addition to elevated T_H_1-stimulating capacity (157). The increased secretion of pro-inflammatory cytokines in these studies suggest that fixation of C1q supports DC maturation and acts in a cC1qR-mediated pathway.

So far, very little data is available on how soluble C1q that is present in the plasma and interstitial tissues under steady state conditions might regulate DC differentiation during the earliest stages of mono-DC growth. These yet unexplored functions would provide important details of how C1q regulates adaptive immune functions via iDCs in the absence of infection or inflammation. Studies from our lab (158) and others (159) have shown that C1q acts as a chemoattractant to iDCs, but not mature DCs. C1q-induced migration is mediated through ligation of both gC1qR and cC1qR and activation of Akt and MAPK pathways. C1q treatment during DC differentiation was also shown to give rise to CD1a^+^DC-SIGN^+^ iDCs with high phagocytic capacity, and low expression of CD80, CD83, and CD86 (154). Because this narrow window of differentiation represents the important interface between innate and adaptive immunity, more work is needed to explore this crucial stage.

## Implications for Therapy and Concluding Remarks

Since C1q and C1qRs are involved in a multitude of inflammatory processes that accompany various disease conditions, including infection, cancer, and autoimmune diseases, understanding the underlying mechanism is important to identify new targets for the design of therapeutic strategies. While the role of C1q in apoptotic clearance has been well described and is supported by a plethora of evidence, it is still not clear how deficiency of C1q contributes to the loss of tolerance. This review is aimed to provide new insights and stimulate discussion around the topic. Understanding how the interactions between C1q and C1qRs control the transition from steady state to a pro-inflammatory response, will not only give us insight into how the C1q/C1qR system regulates the immune response, but may also provide us with alternative approaches for designing better therapeutic options. Molecules or peptides that inhibit the interaction between antigen-bound C1q and cC1qR, or those that can mimic the interaction between C1q and gC1qR, can potentially be used as templates for the development of therapeutic interventions to reduce C1q-mediated pro-inflammatory responses. One potential target for an inhibitory-drug design is the N-terminal region (residues 160–283) on the collagen tail of C1q, which binds to cC1qR, and contains several short (7–10 amino acids) CH2-like motifs (ExKxKx) similar to the C1q binding motif found in the CH2 domain of IgG (160). For gC1qR, some therapeutic molecules already exist. One example is the use of mAb 74.5.2, which inhibits the binding of kininogen to gC1qR, thus blocking the generation of bradykinin and other vasoactive molecules that have been shown to contribute to inflammation (161). Another example of a therapeutic molecule is mAb 60.11, which is specific to the C1q binding site on gC1qR (aa 76–93). This antibody has been shown to reduce cell proliferation, decrease tumor growth, increase apoptosis, and impair angiogenesis (162). In summary, the data reviewed in this article supports the idea that the C1q/C1qR system is an ideal molecular target for the design of antibody- or peptide-based therapy to attenuate acute and chronic inflammation associated with autoimmune diseases, SLE in particular.

## Author Contributions

BG supervised the work. All authors contributed to the article and approved the submitted version.

## Conflict of Interest

The authors declare that the research was conducted in the absence of any commercial or financial relationships that could be construed as a potential conflict of interest.

## Abbreviations

gC1q: the globular heads of C1q
cC1q: the collagen domain of C1q
gC1qR: receptor for gC1q
cC1qR: receptor for cC1q
CR: calreticulin (another name for cC1qR)
ghA, ghB and ghC: globular heads (gh) of the AB and C chains of C1q.
